# Erasmus Syndrome, An Autoimmunity Paradox: A Case Report and Literature Review

**DOI:** 10.7759/cureus.79036

**Published:** 2025-02-15

**Authors:** Alejandro Arango, Simon Villa-Pérez, Jhon Edwar Garcia Rueda, Alejandro Cardona Palacio, Roberto Benavides

**Affiliations:** 1 Internal Medicine, Pontifical Bolivarian University, Medellín, COL; 2 Internal Medicine, Hospital Pablo Tobón Uribe, Medellín, COL; 3 Internal Medicine, Universidad EIA, Envigado, COL; 4 Pathology, Hospital Pablo Tobón Uribe, Medellín, COL; 5 Pathology, Universidad EIA, Envigado, COL; 6 Pulmonology, Universidad CES, Medellín, COL

**Keywords:** erasmus syndrome, ild interstitial lung disease, immunosuppressive agents, silicosis, systemic sclerosis

## Abstract

Erasmus syndrome (ES) is a rare condition characterized by the link between crystalline silica exposure, with or without silicosis, and systemic sclerosis (SSc). Although first noted over a century ago, its underlying mechanisms remain unclear. However, it is indistinguishable from idiopathic SSc in the general population. Its clinical presentation is heterogeneous, depending on the affected systems, with notable features, including skin fibrosis, microstomia, telangiectasia, Raynaud’s phenomenon, arthralgia, and interstitial lung disease. Currently, there is no unified consensus on its treatment; however, organ-specific therapy is a reasonable approach. We report the case of a 43-year-old miner diagnosed with diffuse cutaneous SSc, where ES was diagnosed after an exhaustive history was taken, occupational exposure was characterized, differential diagnoses were excluded, and radiological and histopathological evidence of pulmonary silicosis was presented.

## Introduction

Diffuse interstitial lung diseases (ILDs) are a heterogeneous group of respiratory disorders with multiple etiologies. Among them, the relationship with systemic autoimmune diseases or exposure to certain environmental agents, such as asbestos or crystalline silica, is of great interest [1]. When an ILD is related to an autoimmune process, it can be classified into three categories based on its coexistence with connective tissue disease (CTD): (1) ILD with an established CTD diagnosis; (2) ILD as the initial manifestation of a CTD; and (3) ILD with subtle manifestations suggesting a CTD in the absence of a definitive diagnosis. This third category includes cases that do not meet the full criteria for a specific CTD but present with clinical, serological, or morphological features of autoimmunity. These cases are classified as interstitial pneumonia with autoimmune features, representing an intermediate entity that may evolve into a defined CTD or remain as an isolated ILD with autoimmune characteristics [1,2]. Therefore, the clinical approach of patients with ILD can be challenging, as ILD itself has been proposed as a trigger of autoimmunity, with examples such as Caplan’s syndrome in rheumatoid arthritis or Erasmus syndrome (ES) in systemic sclerosis (SSc) [3]. The latter is characterized by fibrosis of the skin and various organs due to multiple immunological alterations. It is a rare disease, with an incidence of 10-50 cases per million people per year and an approximate prevalence of 40-340 cases per million inhabitants [4]. ES, defined as the association between exposure to crystalline silica, with or without silicosis, and SSc, was first described in 1914 by Bramwell and later documented in 1957 by Erasmus. Although its exact epidemiology is unknown, its prevalence is estimated to be approximately 0.7-0.9% according to the studied cohorts [5,6]. We present the case of a 43-year-old gold miner with a history of inflammatory arthralgias, cutaneous fibrosis, microstomia, Raynaud’s phenomenon, and pulmonary silicosis as the trigger for rapidly progressive diffuse cutaneous SSc.

## Case presentation

A 43-year-old male with a 20-year history as an underground gold miner, who did not use personal protective equipment and had no other relevant exposures or history of toxicity, presented to the emergency department of Hospital Pablo Tobón Uribe with a progressively worsening condition over approximately two years.

The initial manifestation was Raynaud’s phenomenon. Six months later, he developed inflammatory joint pain primarily affecting the hands, wrists, metacarpophalangeal joints, proximal and distal interphalangeal joints, knees, and ankles. After eight to 12 months, he experienced progressive skin thickening and hardening, initially in the hands, leading to restricted digital flexion. A primary care physician attributed these changes to mechanical strain from his occupation.

**Figure 1:**
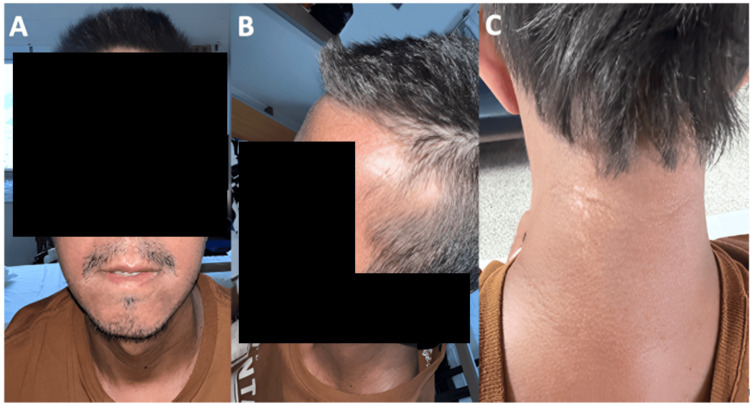
Skin involvement (A) Microstomia. (B) Area of alopecia in the temporal region. (C) Skin induration and salt and pepper lesions.

**Figure 2:**
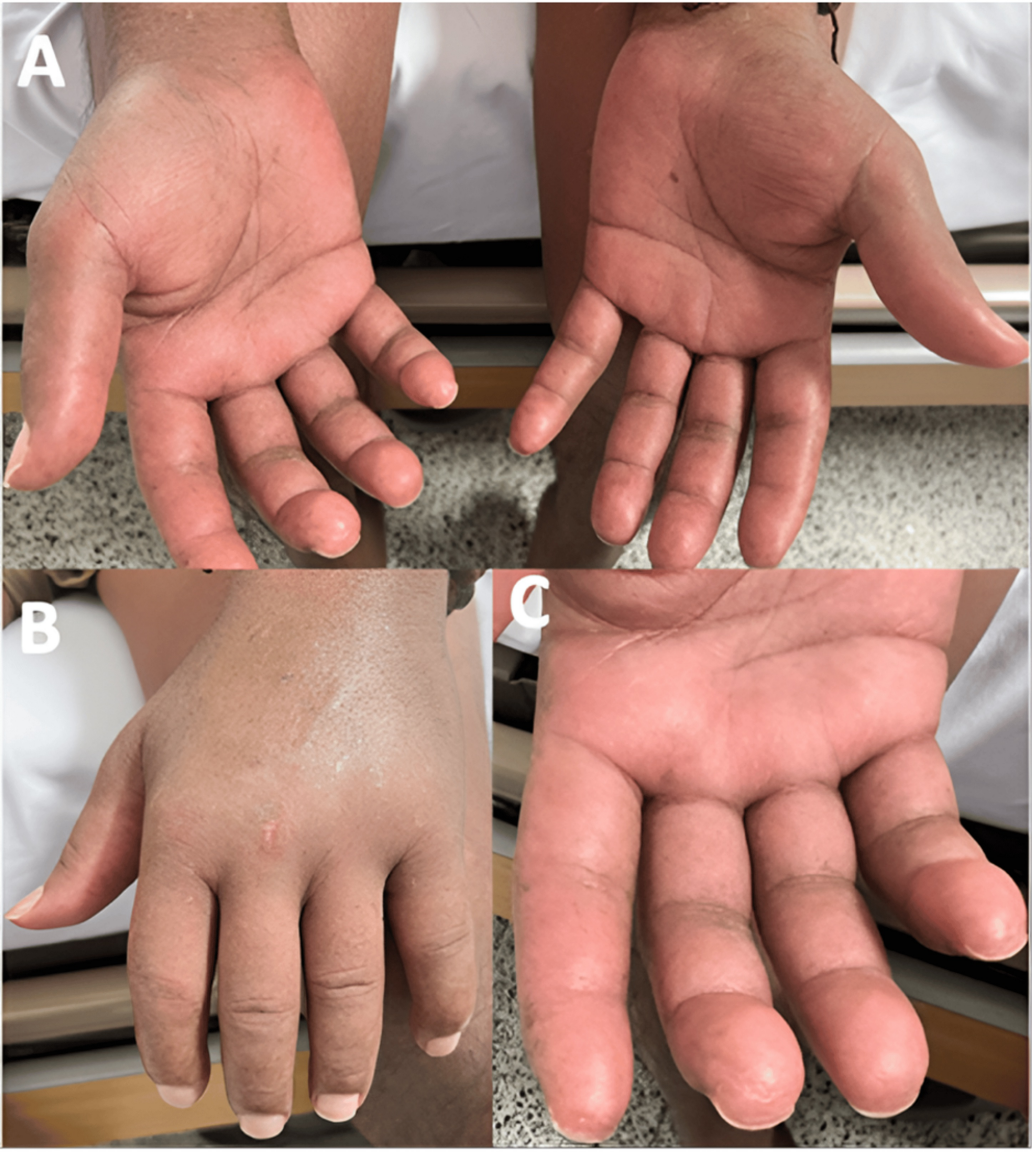
Edematous involvement (A, B) Edema from the wrist to the distal interphalangeal joints, with loss of skin folds on the thumb. (C) Scars from fingertip ulcers.

As the disease advanced, similar skin alterations emerged on his face, neck, and chest, accompanied by increasing difficulty in mouth opening. Over time, he developed fingertip ulcers. He also began experiencing exertional dyspnea, which gradually worsened and initially interfered with his ability to work. However, one month before admission, his dyspnea rapidly progressed to occur with minimal exertion, prompting his visit to our hospital. On physical examination, notable findings included thickened and indurated skin on the hands, forearms, anterior chest, back, neck, and face, with a modified Rodnan skin score of 17 points; telangiectasias on the face; microstomia; hyperpigmented and hypopigmented macular lesions with a “salt and pepper” appearance (Figure 1); “puffy fingers” (Figure 2); healed fingertip ulcers; and pulmonary auscultation revealing bilateral fine inspiratory crackles with a Velcro-like quality.

Considering the clinical presentation highly suggestive of SSc, a series of laboratory tests were ordered (Table 1) to exclude potential mimickers and further support the diagnosis.

**Table 1:** Admission laboratory tests ANA: antinuclear antibodies; anti-CCP: anti-cyclic citrullinated peptide antibodies; C3-C4: complement; RNP: ribonucleoprotein; Scl-70: anti-DNA-topoisomerase I antibody; Sm: Smith antibody; SS-A: Sjögren syndrome antigen A; SS-B: Sjögren syndrome antigen B

Parameter	Patient value	Reference range
Anti-CCP	0.9 U/ml	0-5 U/ml
C3	129 mg/dl	88-201 mg/dl
C4	18.9 mg/dl	15-45 mg/dl
Rheumatoid factor	32.5 IU/ml	0-30 IU/ml
ANA	Homogeneous nuclear pattern at a title of 1:1,280	Negative
Anti-centromere antibodies	<1.0 U/mL	<1.0 U/mL
Anti-DNA antibodies	Negative	Negative
SS-A/Ro	<0.2 U/mL	<25 U/mL
SS-B/La	<0.2 U/mL	<25 U/mL
Sm	<0.2 U/mL	<25 U/mL
RNP	<0.2 U/mL	<25 U/mL
Scl-70	>200 U/ml	<10 U/mL
HIV	Nonreactive	Nonreactive

**Figure 3:**
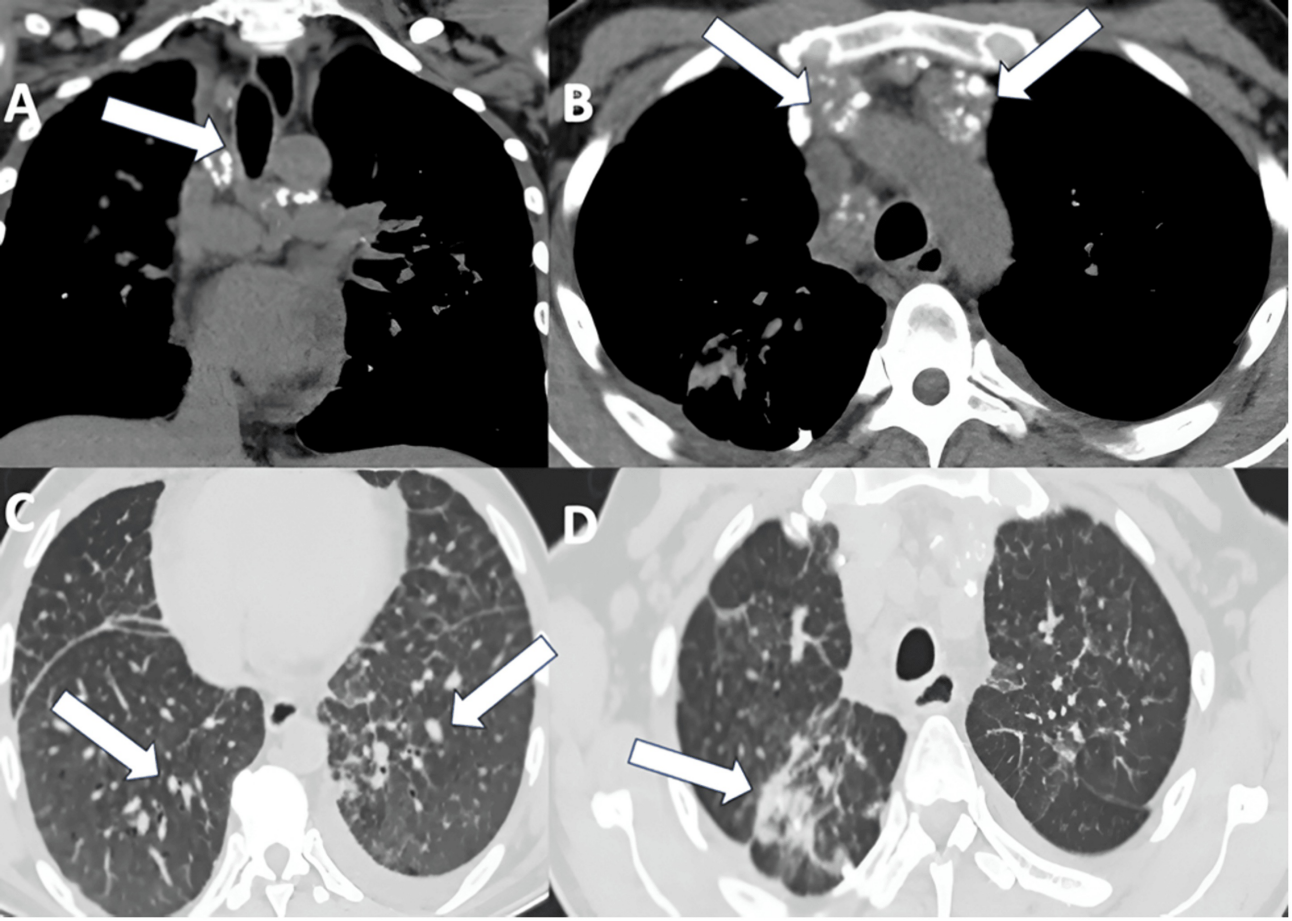
CT chest (A, B) Arrows indicating lymphadenopathy with punctate peripheral calcifications (“eggshell calcifications”). (C) Pulmonary parenchyma with bilateral thickening of interlobular septa and nodular lesions. (D) Pulmonary parenchyma with confluent nodular lesions of irregular morphology in the right upper lobe.

A chest CT scan revealed paratracheal, paraesophageal, subcarinal, and right hilar mediastinal lymphadenopathy, measuring up to 22 mm in size, with punctate and peripheral calcifications. The pulmonary parenchyma exhibited nodular thickening of the interlobular septa and confluent nodular lesions with irregular morphology and calcifications, predominantly affecting the upper lobes bilaterally (Figure 3). An echocardiogram revealed normal size and systolic and diastolic function of the left and right ventricles, with a left ventricular ejection fraction of 62%, longitudinal strain of 19%, and an estimated pulmonary artery systolic pressure of 36 mmHg (maximum tricuspid regurgitation velocity of 289 cm/s).

**Figure 4:**
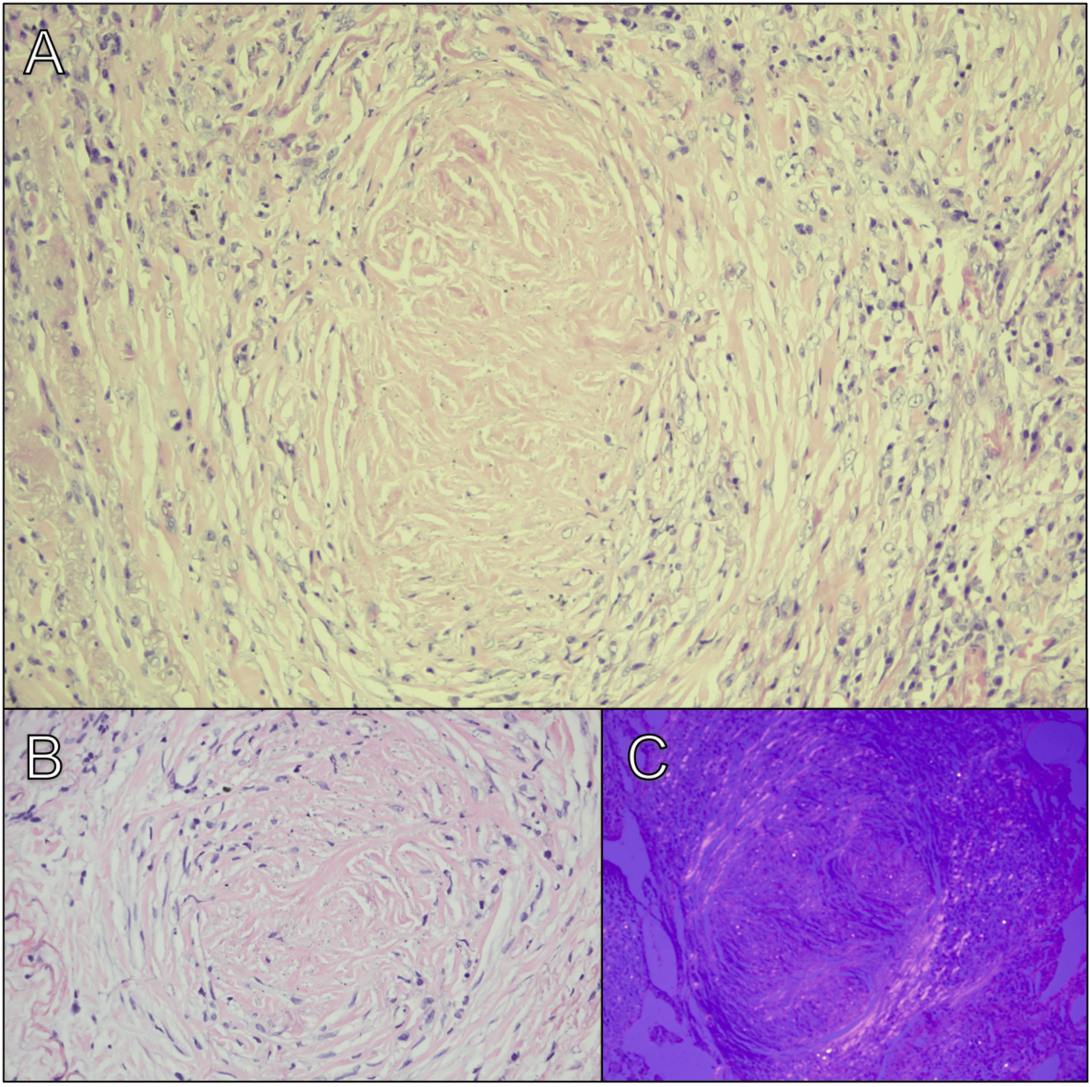
Pathology (A, B) Nodules of fibroblasts and histiocytes with abundant silica. (C) Polarized light shows silica crystals.

Given the discrepancy between the radiological findings and the typical pulmonary involvement in SSc - where ILD usually presents with basal fibrosis and ground-glass opacities - the imaging, in this case, revealed upper lobe-predominant nodular opacities, septal nodular thickening, and irregular nodules forming pseudomass-like lesions. Additionally, mediastinal lymphadenopathy was noted, with characteristic eggshell calcifications. These findings raised suspicion of occupational lung disease, prompting its inclusion in the differential diagnosis. To further assess the nodular lesions and exclude neoplastic and infectious etiologies, a lung biopsy was performed. Histopathological analysis revealed distorted pulmonary parenchyma with nodular aggregates of hyalinized histiocytes and fibroblasts, along with birefringent silica particles under polarized light (Figure 4). No histological features of usual interstitial pneumonia (UIP), nonspecific interstitial pneumonia (NSIP), hypersensitivity pneumonitis, dysplasia, or malignancy were identified. Based on these findings, a diagnosis of silicosis was confirmed as the underlying trigger for secondary SSc. Due to the patient’s rapid disease progression, marked by worsening cutaneous involvement in recent months, intravenous cyclophosphamide was initiated.

## Discussion

Silica can be found abundantly in nature and is released during mining, sandblasting, or the manufacture of artificial stone [7]. It is present in crystalline and amorphous forms with distinct physicochemical properties. Notably, its crystalline form has a polymerized tetrahedral configuration, which confers greater pathogenicity [8]. Prolonged exposure to crystalline silica can result in chronic silicosis after more than 10 years of low-to-moderate exposure, accelerated silicosis within the first 10 years of moderate-to-high exposure, or silicoproteinosis within the first five years following very high levels of silica exposure. Our patient worked as an underground miner in at least four different gold mines over the past 20 years, with six- to eight-hour shifts, five days a week. He never used airway protection, a practice still prevalent in certain regions of South America. Considering the prolonged duration, high-intensity exposure, and absence of respiratory protection, his exposure is classified as severe and chronic (>10 years), placing him at significant risk for progressive pulmonary disease [9]. The pathological mechanism of the silica-lung interaction is complex and not fully understood; however, evidence suggests that lung injury is caused by cytotoxic effects on alveolar macrophages. This occurs through the activation of surface receptors and the induction of reactive oxygen species, leading to cellular damage [10].

In ES, pulmonary involvement arises from immunological alterations triggered by the pathogenic effects of crystalline silica. This stimulates the production of rheumatoid factor, hypergammaglobulinemia, functional abnormalities in T lymphocytes, and soluble IL-2 receptors, which in turn lead to increased production of IL-1, IL-6, interferon gamma, and tumor necrosis factor alpha. This cascade promotes fibroblast production, collagen deposition in the extracellular matrix, and subsequent fibrosis in the skin and lungs, as well as vascular occlusion. In the lungs, this process primarily manifests as ILD, with UIP and NSIP being the most common patterns. NSIP is more frequently observed in SSc and is characterized by ground-glass opacities and fibrosis predominantly in the lower lobes, whereas UIP, although less common, is associated with a worse prognosis due to its progressive fibrotic nature [11-13].

Physiologically, when silica particles are phagocytosed by alveolar macrophages, an acute and chronic inflammatory niche is formed, completely negating the immune function of these cells, which explains the predisposition of these individuals to coinfections with *Mycobacterium tuberculosis *[14]. This process can be explained by the activation of multiple inflammatory pathways, particularly the NLRP3 inflammasome. Upon binding to the apoptosis-associated speck-like protein adapter, NLRP3 activates caspase-1, which cleaves the immature proforms of IL-1 beta, IL-18, and IL-33 into their mature (highly inflammatory) forms, thereby amplifying the activity of HMGB1 and certain heat shock proteins that facilitate leukocyte transmigration into the pulmonary interstitium [15].

Other theories propose that the production of free radicals by alveolar macrophages leads to damage to type I pneumocytes, causing proliferation of type II pneumocytes and increased recruitment of inflammatory cells, thereby amplifying cellular damage [13].

Finally, Haustein and Anderegg [16] documented microvascular activation induced by crystalline silica, followed by endothelial changes, infiltration of mononuclear cells, and fibroblasts in a manner similar to the pathophysiological process in patients with idiopathic SSc, thereby highlighting the lung as a key trigger for systemic autoimmunity [16].

Among ES cohorts, antinuclear antibodies positivity has been reported in 100% of individuals, with a predominance of speckled or homogeneous patterns; anti-topoisomerase I antibodies are present in 44% of cases; and various HLA-DQB alleles, particularly subtypes 0506, 0305, and 0303, have been identified in some patients [17]. Similarly, studies such as that conducted by Rustin et al. [18] have convincingly demonstrated that individuals who develop SSc following significant silica exposure express anti-topoisomerase I antibodies that are indistinguishable from those found in the idiopathic SSc population [18].

The clinical presentation is heterogeneous and depends on the affected population, occupational exposure, comorbidities, and extent of pulmonary involvement [19]. Raynaud’s phenomenon is frequently described in the literature in up to 85% of cases, often as a sentinel event (occurring even five years prior to the onset of SSc symptoms). Digital ulcers occur in 48% of cases, ILD in 78%, and silicosis in up to 40% of cases [19]. Other manifestations commonly associated with SSc include skin thickening, sclerodactyly, microstomia, puffy fingers, telangiectasia, acro-osteolysis, or calcinosis cutis [13,14].

With respect to risk factors, Rocha et al. [17] evaluated a population of 947 subjects treated at two referral centers in Brazil between 2000 and 2014, focusing on variables such as age, sex, age at onset of SSc, duration of silica exposure, and the extent of skin and lung involvement [17]. They concluded that ES was strongly associated with the accumulated time of silica exposure, with a mean duration of 13.7 years, predominantly affecting males (78%), a Rodnan skin score greater than 20, and an average age of onset of 47 years [17].

The management of ES is guided by the extent of organ involvement secondary to scleroderma. Given the absence of specific treatment recommendations in international guidelines, therapeutic approaches are primarily based on case reports. Calcium channel blockers are used for Raynaud’s phenomenon and methotrexate for musculoskeletal manifestations. Cyclophosphamide could be considered in cases of rapidly progressive disease; however, due to the rarity of ES and the lack of robust evidence, a strong recommendation cannot be made [20].

## Conclusions

We report the case of a man with significant occupational exposure to crystalline silica, followed by the development of diffuse cutaneous SSc and pulmonary silicosis. A thorough history and physical examination are crucial in establishing the relationships among occupational exposure, pulmonary silicosis, and systemic autoimmunity. This case highlights the importance of the lung as a key pathophysiological site in rheumatologic disease. Additionally, a comprehensive literature review revealed no specific treatment guidelines for ES across major international recommendations. The available evidence is limited to case reports, making therapeutic decisions largely extrapolated from idiopathic SSc management. In patients with ILD or rapidly progressing cutaneous involvement, cyclophosphamide could be a reasonable treatment option.
